# Effect of Fluid Contamination on Reverse Torque of Healing Abutments in Two Implant Connection Systems: In Vitro Study

**DOI:** 10.1111/cid.70056

**Published:** 2025-05-26

**Authors:** Shifra Levartovsky, Guy Ronen, Perry Raz, Ilan Beitlitum

**Affiliations:** ^1^ Department of Oral Rehabilitation, the Maurice and Gabriela Goldschleger School of Dental Medicine Tel Aviv University Tel Aviv Israel; ^2^ Department of Periodontology and Dental Implantology, the Maurice and Gabriela Goldschleger School of Dental Medicine Tel Aviv University Tel Aviv Israel

**Keywords:** conical connection, contamination, healing abutment, internal hex connection, reverse torque

## Abstract

**Introduction:**

This in vitro study investigates the impact of blood, saliva, and chlorhexidine contamination on the reverse torque values (RTVs) of healing abutments in two types of implant‐abutment connections: internal hex and conical.

**Methods:**

A total of 88 Ti6Al4V titanium alloy dental implants were tested, comprising two types of connections: internal hex (*n* = 44, MIS Seven) and conical (*n* = 44, MIS C1). Each group was further divided into four subgroups (*n* = 11) based on the type of contamination medium used. Healing abutments were tightened onto the implant fixtures using a torque of 25 Ncm with the designated medium, followed by retightening after a 10‐min interval. After a two‐week incubation period, reverse torque values (RTVs) were recorded using a digital torque gauge. To assess differences between the implant systems within the same medium, as well as differences between the medium types within the same implant system, the Welch two‐sample *t*‐test was employed. Additionally, linear regression analysis was conducted to evaluate the interaction between medium types and the implant systems.

**Results:**

For MIS Seven implants, RTVs were consistently lower than the initial 25 Ncm torque across all contamination groups. In contrast, for MIS C1 implants, RTVs exceeded 25 Ncm in the saliva and blood contamination groups; however, the presence of chlorhexidine reduced the RTVs. A comparative analysis revealed that the C1 conical connection required significantly higher RTVs than the Seven internal hex system (coefficient = 3.318). No significant differences were observed between implant systems in the control and chlorhexidine groups. However, the C1 system required higher RTVs than the Seven system in the presence of saliva (*p* = 0.0006) and blood (*p* = 0.00009). Furthermore, the interaction analysis indicated that in the presence of saliva and blood contamination, C1 implants required significantly higher RTVs, with mean differences of 7.18 and 4.9 (Ncm), respectively, compared to the Seven implants.

**Conclusions:**

Conical implant‐abutment connections generally require higher RTVs for abutment disconnection compared to internal hex connections. Both implant connection and the type of contaminant significantly affect RTVs. Chlorhexidine has been shown to reduce reverse torque for both types of connections. In contrast, contamination with saliva and blood tends to increase reverse torque, particularly for conical connections.

## 1 Introduction

In recent years, the success rate of dental implants has exceeded 90%, largely attributed to significant advancements in implant design, surgical techniques, and growing clinical expertise in the field [1]. Dental implant connections are broadly categorized into external and internal types. External connections feature an interface above the implant platform but may allow micromovement at the implant‐abutment junction, increasing the risk of biological and mechanical complications [2, 3, 4].

Internal connections, including flat‐to‐flat systems (e.g., hexagons) and conical connections, reduce screw loosening, improve force distribution, and minimize implant‐abutment gaps, limiting bacterial penetration in vitro [5, 6, 7, 8]. Conical connections secure the abutment to the implant via a machine taper, where a conical male component fits into a matching female socket, locking through mechanical friction.

Despite advancements, mechanical and biological complications persist, with screw loosening being the most common mechanical issue, affecting 2%–45% of abutments [9, 10]. Studies report that 26% of screws require retightening within the first year [11], with loosening rates of 3.1%–10.8% over 5 years [12]. Notably, these studies did not specify the type of implant connection used.

Screw loosening occurs when the clamping force, generated by the elastic recovery of the implant to maintain abutment contact, is insufficient to counteract separating forces [13, 14]. If the separating force exceeds the clamping force, the screw may loosen, compromising the implant system. When tightened with torque, a screw elongates, creating tension (preload) that determines the clamping force [13, 15]. Preload is influenced by factors such as screw design, material, applied torque, delivery system, fluid contamination, lubrication, and the settling effect (embedment relaxation) due to surface roughness from manufacturing [13, 16, 17, 18, 19]. During tightening, these rough surfaces flatten, reducing preload by 2%–10%, explaining why removal torque is lower than initial tightening torque [13, 18].

Healing abutments are often overlooked in implant dentistry, despite loosening being a common complication. This can cause gum sensitivity, hyperplasia, inflammation, patient discomfort, and increased costs. Loosening also creates a micro‐gap at the implant‐abutment interface, promoting micro‐leakage and biological complications [20, 21].

Conversely, a less common but more serious issue is a healing abutment that cannot be loosened, even with a ratchet tool. High reverse torque may be required, risking implant osseointegration, especially in augmented ridges, and potentially rendering the implant unusable [22].

Studies show comparable outcomes for submerged and non‐submerged implants [23, 24]. Therefore, the placement of the healing abutment is determined by the surgeon based on several factors, including the torque measured during implant insertion and the patient's local and systemic risk factors. Once attached, healing abutments are subjected to external loads (e.g., food) and internal forces (e.g., tongue pressure). In clinical practice, surgeons typically tighten healing abutments manually without using a torque meter. However, the manufacturer's guidelines recommend tightening the healing abutment with a ratchet to a torque of 25 Ncm for MIS implants.

Using the manufacturer's recommended torque during healing abutment tightening stabilizes the screw joint and ensures future removal, but torque values are often unspecified for healing abutments. Repeated insertion and removal of components like cover screws, healing abutments, and impression parts during clinical practice expose the implant‐abutment connection to fluids (e.g., blood, saliva, chlorhexidine) [10, 20]. This contamination alters the friction coefficient, potentially reducing preload and increasing the risk of screw loosening [25].

Research shows that saliva contamination can increase preload, affecting mechanical stability, and highlights the importance of removing contaminants during implant customization to prevent screw loosening [26]. While Duarte et al. [27] reported that fluoridated artificial saliva raises reverse torque values (RTVs), Micarelli et al. [26] found no significant effect of chlorhexidine gel.

Adawi et al. demonstrated that blood contamination significantly reduces RTVs compared to uncontaminated controls. In a survey of 210 implantologists, 80% expressed concern about blood contamination, particularly before abutment loading, with most using chlorhexidine or saline for decontamination. The study found that irrigating with 5.25% sodium hypochlorite followed by 0.12% chlorhexidine gluconate effectively reduced contamination while preserving torque [28]. Similarly, Gumus et al. [20] reported reduced RTVs after contamination with chlorhexidine, saliva, or blood.

The research conducted so far has primarily focused on the significant issue of opening and closing torque values of abutments, as well as the impact of external contaminant fluids on the opening torque of abutments under loading during use. However, there has been limited research addressing the interim period between 1‐stage, non‐submerged implantation and the connection of the abutment and loading of the implant, during which the implant is covered by a healing abutment. Moreover, most of the aforementioned studies have focused on the effects of different mediums on the RTVs of internal hex connection implants [20, 26, 27, 28]. Prado et al. [25] found that biofilm lubrication reduces RTVs in Morse taper (MT) connections, while Sammour et al. [29] showed that dynamic cyclic loading resulted in better screw stability for conical connections compared to internal hex systems. To our knowledge, no study has compared RTVs across different mediums between internal hex and conical connections.

Thus, our study examines the impact of blood, saliva, and chlorhexidine contamination on the RTVs of healing abutments in two implant‐abutment connection types—internal hex and conical—using an in vitro model. The null hypothesis suggests no statistically significant difference in RTVs between implant groups exposed to different contaminants at the implant‐abutment interface.

## 2 Materials and Methods

### 2.1 Tested Implants and Healing Abutments

**FIGURE 1:**
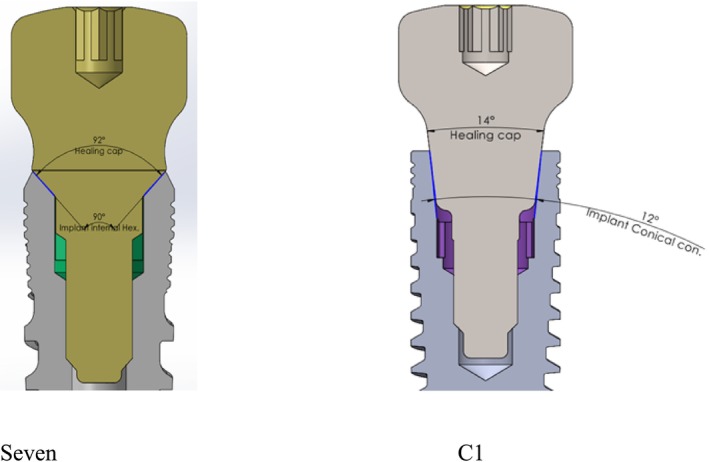
Cross‐sectional view of the healing abutment connections for Seven and C1 implants. The blue lines indicate the contact area between the abutment and the implant.

Eighty‐eight Ti6Al4V titanium alloy dental implants (MIS Implants Technologies Ltd., Bar Lev Industrial Park, Misgav, Israel) with an internal hex connection (*n* = 44, MIS Seven) and conical connection (*n* = 44, MIS C1) were tested (Figure 1).

All implants (3.75 × 11.5 mm) featured a tapered, dual‐thread design and underwent the same surface treatment (sandblasting and acid etching). The internal conical connection has a 2 mm depth and a 12° cone angle. The implants were paired with compatible standard healing abutments (Ø4.8 mm, *H* = 3 mm, MH‐A3375, CS‐HS348, MIS).

Each implant group (internal hexagon or conical connection) was divided into four subgroups (*n* = 11) based on the contamination medium: a control group (no medium), a fresh saliva group (collected from a donor at rest), a blood group (obtained via fingertip lancet from the same donor), and a 2% chlorhexidine gel group (Corsodyl gel, Haleon, Weybridge, UK). Before mechanical testing, the implant's inner chamber was filled with the designated medium using a pipette to fully coat the inner surface. The healing abutment was then inserted into the implant body. The mechanical test aimed to evaluate the settling effect and torque loss following the initial tightening.

### 2.2 Implants Fixation

**FIGURE 2:**
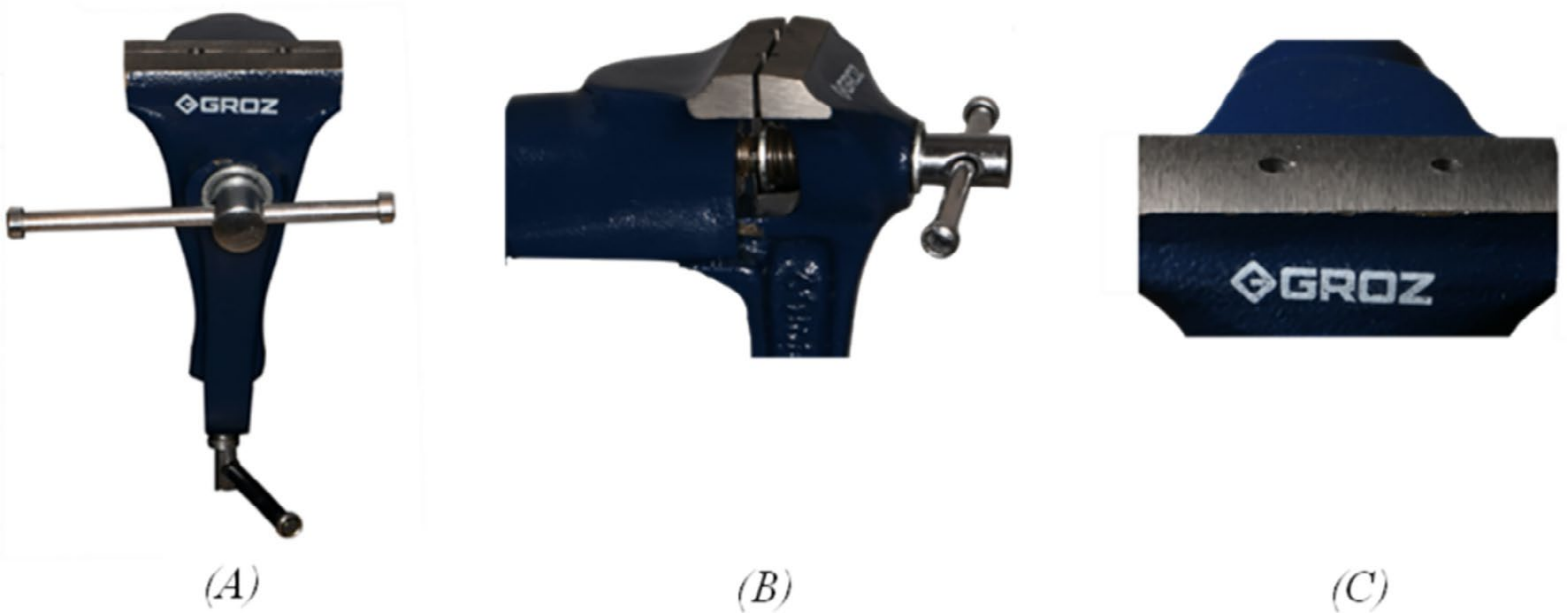
The modified Groz clamp with drilled chambers of specified dimensions. (A) Frontal view; (B) side view; (C) close‐up view.

To measure the closing and opening torque of the healing abutments, implant immobilization during force application was essential. To achieve this, two drilled holes (3.5/10.5 mm) were made in a clamp (Groz, TBV/C/50, USA) to ensure standardized positioning (Figure 2).

**FIGURE 3:**
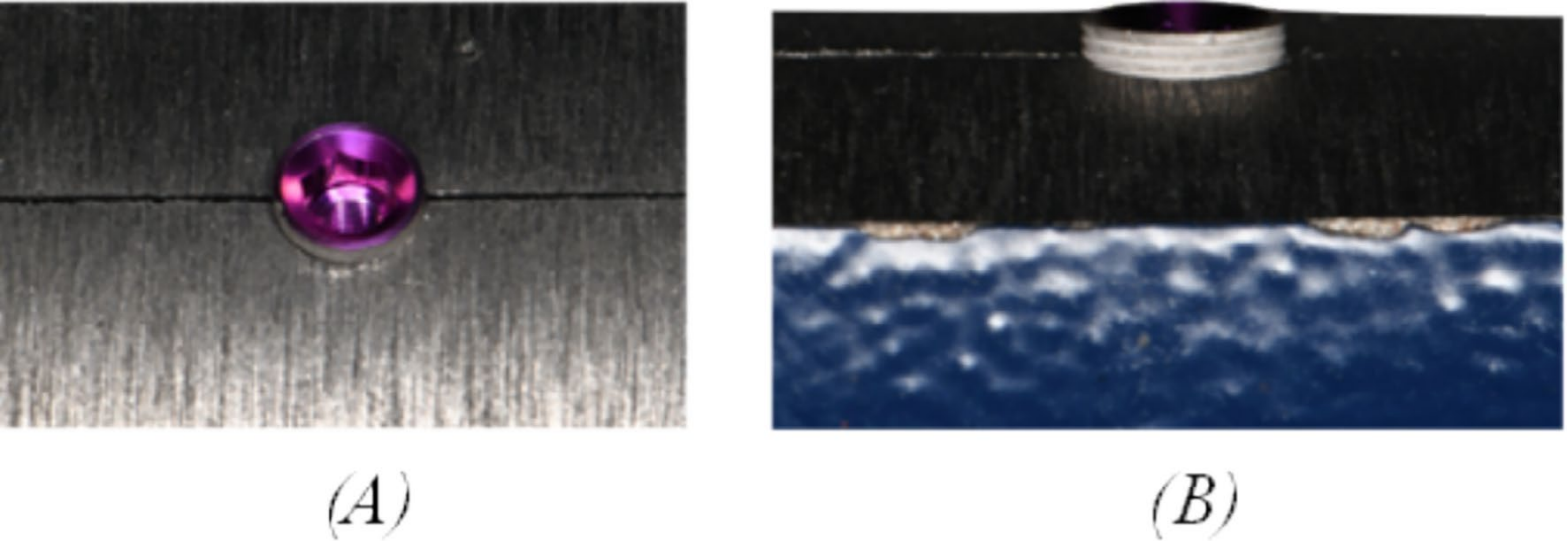
MIS Seven implant secured in the customized drill. (A) Top view; (B) side view.

The clamp was securely fixed to a table, and each implant was inserted into its corresponding hole. The clamp was then tightened to immobilize the implants, leaving 1 mm of the implant collar exposed above the chamber (Figure 3).

### 2.3 Healing Abutment Tightening

**FIGURE 4:**
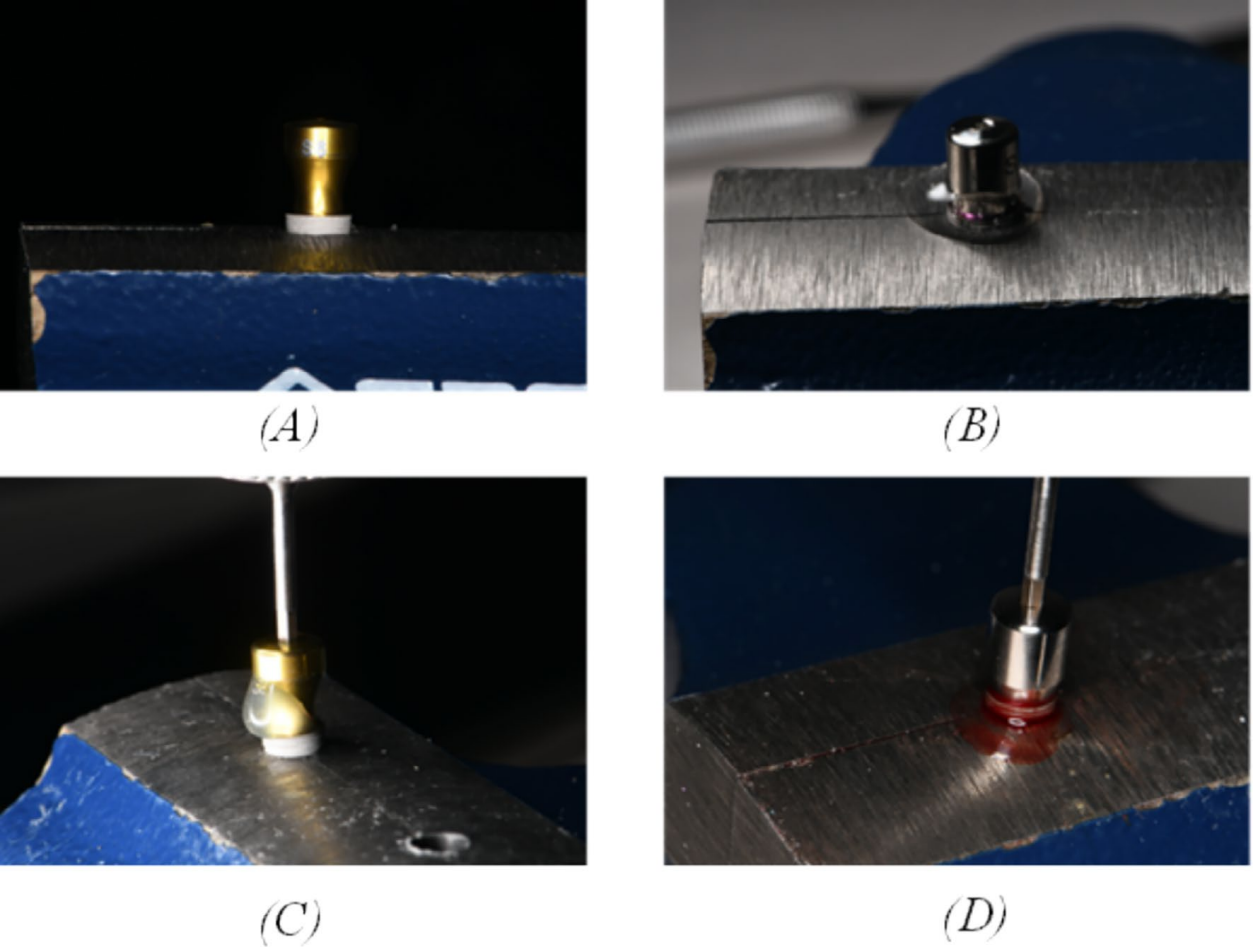
(A) C1, control group; (B) Seven, chlorhexidine group; (C) C1, saliva group; (D) Seven, blood group.

The healing abutments were tightened onto the implant fixture with the designated medium using a torque of 25 Ncm, as per the manufacturer's recommendation (Figure 4).

**FIGURE 5:**
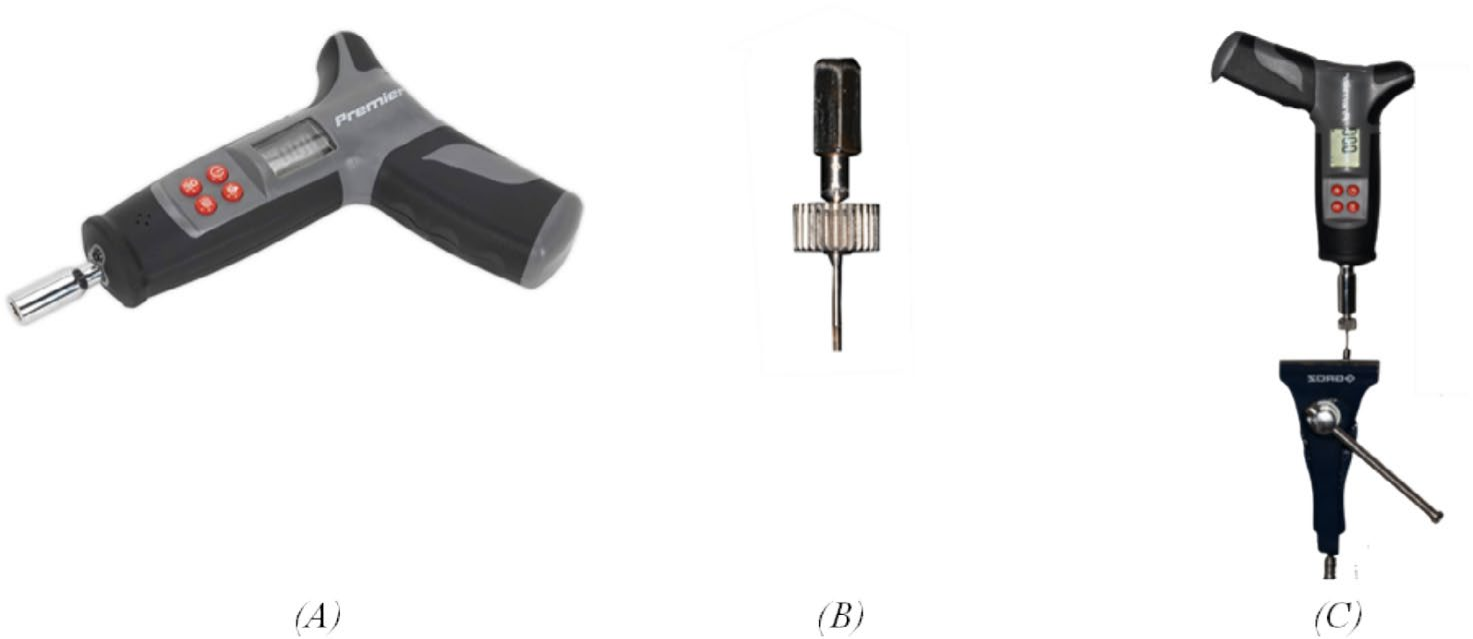
(A) Premier digital torque gauge; (B) implant driver attached to a customized adaptor; (C) torque gauge connected to the implant driver, securing the healing abutment mounted in the Groz clamp.

This was accomplished using a digital torque gauge (STS104 Digital Torque Screwdriver, Premier, UK) fitted with a 0.05‐in. hex driver attached to a customized adapter (Figure 5).

After a 10‐min interval, the healing abutments were retightened to a 25 Ncm torque, consistent with the initial torque. This step was performed to compensate for any settling effects that may have occurred.

Samples were incubated in 50 mL tubes containing artificial saliva (Biotène Weybridge, England, UK) in an incubator at 37°C for 2 weeks, trying to imitate oral cavity conditions.

### 2.4 Reverse Torque Values Measurements

After the two‐week incubation period, reverse torque measurements were recorded during the removal of the healing abutment using the same digital torque gauge (STS104 Digital Torque Screwdriver, Premier, UK) configured in reverse mode. The digital torque meter was not set to a threshold value during the removal process. The digital display gradually showed an increase in torque until it reached the peak value at the moment of the healing abutment's disconnection, after which it rapidly decreased, as expected. This peak value was recorded as the removal torque value (RTV).

### 2.5 Statistical Analysis

A two‐way ANOVA was used to compare RTVs across different contamination fluid groups and implant connection systems. The Welch two‐sample *t*‐test was applied to assess differences between implant systems within the same medium and between mediums within the same implant system. Additionally, linear regression analysis was performed to evaluate the interaction between medium types and implant systems. A significance level of 5% (*p* < 0.05) was set for all analyses to indicate statistical significance.

## 3 Results

Descriptive statistics and reverse torque values (RTVs) for all groups are summarized in Table 1.

**TABLE 1:** RTVs (Ncm) (mean ± SD) during the removal of the healing abutments for each medium tested.

	*N*	Mean	SD	Min	Max	*p*	RTVs lost/added (%)
Seven							
Control	11	24.72	2.28	23	30		−1
Chlorhexidine	11	22.09	2.91	19	28	0.0029	−12
Saliva	11	24.55	1.29	22	26	0.821	−2
Blood	11	22.64	2.41	19	27	0.05	−9
C1							
Control	11	25.45	3.05	22	31		+2
Chlorhexidine	11	21.09	1.38	19	24	0.006	−16
Saliva	11	30.18	3.92	26	40	0.005	+21
Blood	11	30.55	4.39	25	41	0.005	+22

Abbreviations: Max, maximum; Min, minimum; RTVs, reverse torque values; SD, standard deviation.

**FIGURE 6:**
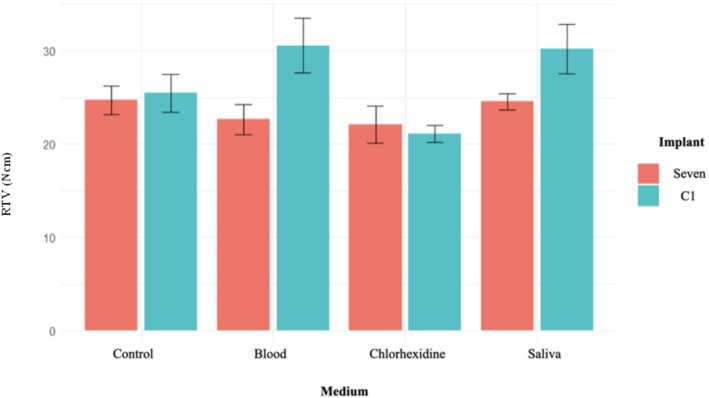
Mean RTV (Ncm) with confidence intervals, categorized by contamination group and implant type.

The results demonstrated that RTVs varied depending on the implant system (Figure 6).

For MIS Seven implants with an internal hex connection, RTVs were consistently lower than the initial closing torque of 25 Ncm across all contamination groups. The highest mean RTV, 24.72 ± 2.28 Ncm, was observed in the non‐contaminated control group. Contamination with chlorhexidine, saliva, and blood resulted in reduced mean RTVs of 22.09 ± 2.91, 24.55 ± 1.29, and 22.64 ± 2.41 Ncm, respectively.

In contrast, for MIS C1 implants with a conical implant‐abutment connection, mean RTVs exceeded the initial 25 Ncm closing torque in the saliva (30.18 ± 3.92 Ncm) and blood (30.55 ± 4.39 Ncm) contamination groups. However, the chlorhexidine group showed a lower mean RTV of 21.09 ± 1.38 Ncm.

Therefore, in the Seven group, all samples exhibited a loss of mean RTVs, while in the C1 group, most samples showed an increase in mean RTVs, except for those in the Chlorhexidine medium.

**FIGURE 7:**
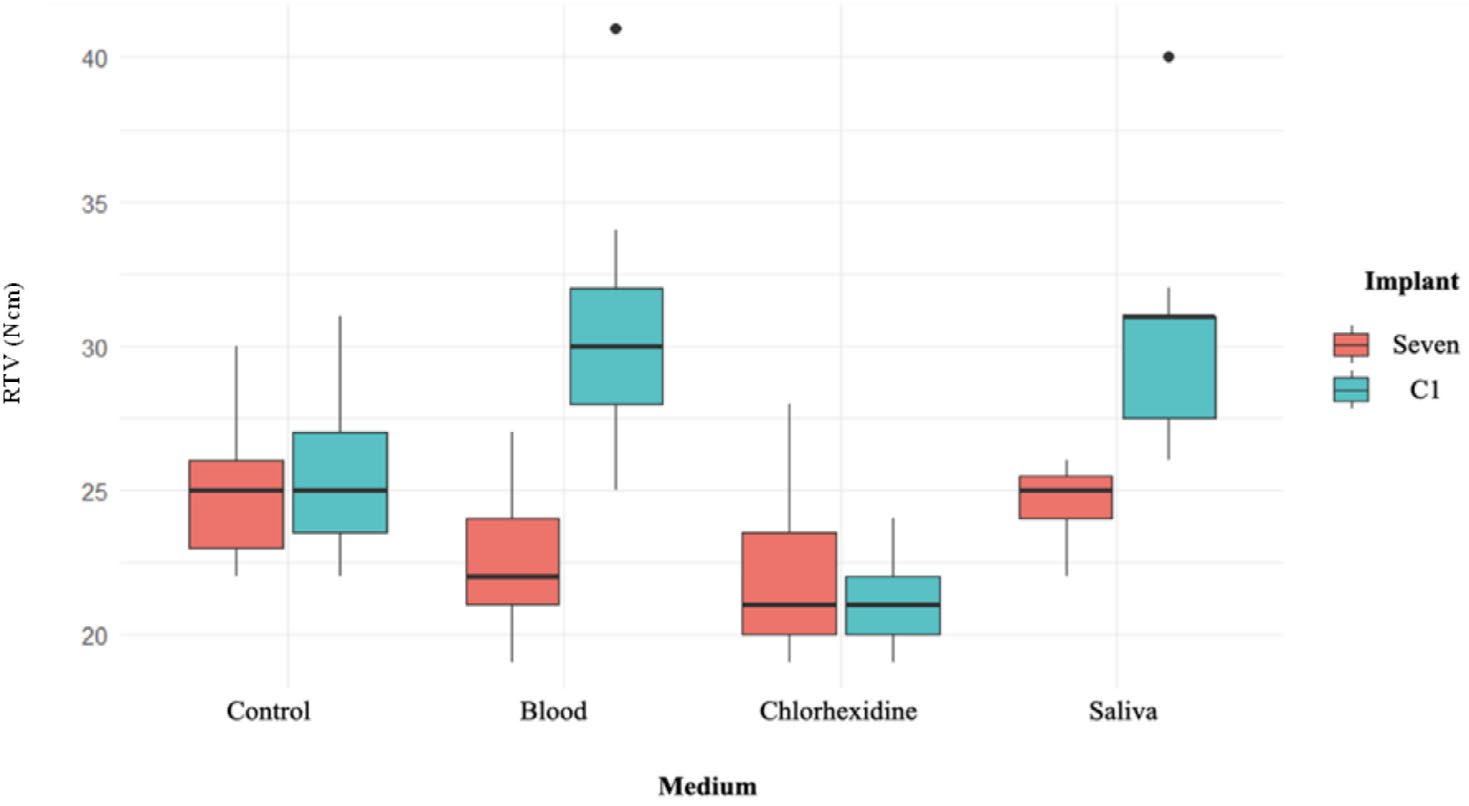
Box plot illustrating the mean RTVs (Ncm) for each implant system within the same contamination group.

Comparative analysis showed that, regardless of contamination, the C1 conical connection system required significantly higher mean RTVs compared to the Seven internal hex system, with a positive coefficient of 3.318. When analyzed by contamination group, no significant difference in mean RTVs was observed between the Seven and C1 implants in the control and chlorhexidine groups. However, the C1 system required significantly higher mean RTVs than the Seven system in the presence of saliva (*p* = 0.0006) and blood (*p* = 0.00009) contamination (Figure 7).

Within each implant system, contamination had distinct effects. For MIS Seven implants, the chlorhexidine group showed a significantly lower mean RTVs compared to the control group, while saliva and blood contamination had no significant impact on RTVs. In contrast, for MIS C1 implants, both blood and saliva contamination significantly increased mean RTVs compared to the control group, whereas chlorhexidine contamination significantly reduced the mean RTVs.

The linear regression analysis of the interaction between medium types and implant systems revealed no significant difference in the mean RTVs between the Seven and C1 implants in the control and chlorhexidine groups. However, in the presence of blood and saliva contamination, the C1 implants required significantly higher mean RTVs, with mean differences of 7.18 and 4.9 (Ncm), respectively, compared to the Seven implants (Table 2).

**TABLE 2:** Linear regression‐implant effect. Mean RTV difference between C1 versus Seven.

	RTV (Ncm)	*p*	
Control			
Chlorhexidine	3.31	1.57E‐05	***
Blood	1.5	0.14	NS
Chlorhexidine	−3.5	9.60E‐04	***
Saliva	2.27	0.029	*
Intercept	23.43		

*** *p* < 0.001.

* *p* < 0.01.

## 4 Discussion

This study evaluated the impact of blood, saliva, and chlorhexidine contamination on the RTVs of healing abutments in two implant‐abutment connection types—internal hex and conical—using an in vitro model. This study primarily compared a 12° conical connection with an internal hex connection. The conical connection relies on friction between a conical pillar and cavity, with friction increasing due to their parallelism. The connection angle depends on the material's mechanical properties, influencing friction. In contrast, the internal connection has minimal surface interaction between abutments and implants, relying instead on the implant's top line and the screw's clamping force (Figure 1). Notably, the contact area in the C1 implant is significantly greater and more parallel compared to the Seven implant.

Repeated tightening of the healing abutment can significantly impact the preload of the final prosthetic abutment. This is an important mechanical consideration that requires attention. Clinically, this suggests that the recommended torque value for the final prosthetic abutment may need to be adjusted if the implant has undergone multiple tightening cycles of the healing abutment during the treatment process.

Our null hypothesis was rejected, as statistically significant differences in RTVs were observed between the implant groups exposed to different contaminants at the implant‐abutment interface.

Mechanical factors such as implant‐abutment connection fit and abutment screw preload are critical in implant rehabilitation. The implant's dimensions and geometry significantly influence stress distribution and magnitude. Sammour et al. compared abutment screw torque loss ratios before and after cyclic loading between internal hex and conical connections. Their findings indicated that conical connections exhibited higher post‐load torque values due to cold welding at the implant‐abutment interface [29].

The use of the same alloy for both healing abutments and implants is a key factor in interpreting the results. MIS dental implants and healing caps are manufactured from the same titanium alloy (Ti‐6Al‐4V, 90% titanium, 6% aluminum, 4% vanadium), ensuring material consistency across groups and eliminating alloy composition as a confounding factor. In contrast, implant systems using a softer implant alloy than the abutments might yield different results.

In conical connections, tightening torque is influenced by screw height and the wedge effect caused by the conical abutment's sinking. The load is primarily supported by the internal fixture slope, reducing stress on the abutment screws compared to external butt joint systems. The cold‐welding effect inherent in conical designs enhances torque gain through friction between the conical abutment and internal implant surfaces, providing exceptional stability [30]. This results in superior sealing, reduced micro‐gap formation, improved torque maintenance, and greater abutment stability [31]. However, the cold‐welding effect may make a healing abutment irretrievable, even with a ratchet tool, risking damage to the implant [22].

Caballero et al. [32] evaluated the mechanical behavior of five MT connection designs with cone angulations of 11.5°, 15°, and 16°, demonstrating that lower cone angulation increases friction between components. Smaller cone angles enhance the wedge effect due to greater sinking of the conical abutment. Similarly, de Freitas et al. [33] studied three taper angles—11.5°, 16°, and 24°—and found that smaller angles in Morse‐type connections result in greater axial displacement of the prosthetic abutment.

The current study compared implants with an internal hex connection (MIS Seven) and a conical connection (MIS C1). The MIS C1 implants, featuring a 2 mm deep internal conical connection with a 12° cone angle, demonstrated a stronger wedge effect, potentially influencing the RTVs of healing abutments. Consistently, we found that, regardless of contamination, the C1 conical connection required significantly higher RTVs than the Seven internal hex system, although the healing abutment has a 14° conus, with a positive coefficient of 3.318.

These findings align with those of Sammour et al. [29], who reported that conical hybrid connections exhibited the lowest percentage of initial and post‐load torque loss compared to internal hex connections, with statistically significant differences. They also observed that the conical connection's post‐load torque value exceeded initial torque due to cold welding at the implant‐abutment interface. Similarly, in our study, the MIS C1 implants with a conical connection showed RTVs surpassing the initial 25 Ncm closing torque in saliva and blood contamination conditions.

The current study also investigated the impact of contamination on RTVs. For both implant types, the chlorhexidine group exhibited significantly lower RTVs compared to the control group. These findings are consistent with those of Shemtov‐Yona et al. [34], who observed notable torque loss when opening abutment screws in chlorhexidine and fluoride mouthwash mediums after dynamic loading, whereas blood and saliva required higher torque. In our study, saliva and blood contamination had no significant effect on RTVs in the internal hex group. However, in the conical connection group, both blood and saliva contamination significantly increased RTVs compared to the control group. These findings have clinical implications, particularly for implants with conical connections, as excessive force may be required to retrieve healing abutments seated in blood or saliva mediums. A potential solution is to immerse healing abutments in chlorhexidine gel prior to placement to address this issue. The placement of the implant‐abutment connection subgingivally raises an important consideration regarding the potential effects of gingival crevicular fluid (GCF) on the mechanics of the connection. The presence of GCF creates a dynamic biological environment, making the mechanical behavior of the connection less predictable than in controlled laboratory settings. Therefore, this biological factor must be considered when evaluating torque values and connection stability in clinical situations. Unfortunately, most in vitro studies are unable to fully replicate the complex biochemical environment created by GCF.

## 5 Limitations and Strengths

### 5.1 Strengths

To the best of our knowledge, this is the first study that compares the removal torque values of healing abutments across different mediums between two types of implant‐abutment connections: internal hex and conical connections.

### 5.2 Limitations

The limitations of this study include its in vitro design, which cannot fully replicate the complexities of the intraoral environment. While the lack of dynamic loading is a limitation, it is less significant for healing abutments due to their small height, which reduces their exposure to dynamic loading in the oral environment. Additionally, since only one time frame was evaluated, we cannot conclusively determine the impact of varying waiting periods on blood clot stability and removal torque values. A two‐week period, typically recommended for tissue maturation in a two‐stage approach, was chosen for this study, though longer waiting may increase RTV. Finally, evaluating only one type of contamination at a time does not fully represent clinical scenarios, where multiple types may occur simultaneously.

## 6 Conclusions

1. Implants with a conical implant‐abutment connection generally require higher reverse torque values for healing abutment disconnection compared to internal hex connection implants.
2. Both implant connection design and contaminant type have a significant interactive effect on reverse torque values.
3. Chlorhexidine, an antimicrobial agent, appears to reduce the required reverse torque values for both implant connection types.
4. Contamination of the implant‐abutment interface with saliva or blood increases reverse torque values, particularly for conical connection implants. Therefore, it is essential to thoroughly rinse the implant head connection before inserting the abutment to minimize this risk.

## Author Contributions

**Shifra Levartovsky:** design, data collection, analysis and article writing. **Guy Ronen:** data collection, analysis, revision and approval. **Perry Raz:** data collection, statistics and analysis. **Ilan Beitlitum:** concept, data analysis, statistics and article writing.

## Ethics Statement

Ethical approval was obtained from Tel‐Aviv University Ethics Committee (0007697‐1).

## Consent

The donor provided signed informed consent to participate in the study.

## Conflicts of Interest

The authors declare no conflicts of interest.

## Data Availability

The data that support the findings of this study are available on request from the corresponding author. The data is not publicly available due to privacy or ethical restrictions.
